# Assigned vs. observed relative age: the association of early entry to school on cognitive abilities and its implications for educational practice

**DOI:** 10.3389/fpsyg.2026.1708843

**Published:** 2026-02-18

**Authors:** Ingo Roden, Nick Wattie, Stephan Bongard, Jörg Schorer

**Affiliations:** 1 Department of Educational Psychology, Carl von Ossietzky University Oldenburg, Oldenburg, Germany; 2 Institute of Technology, University of Ontario, Oshawa, ON, Canada; 3 Department of Psychology, Goethe-University Frankfurt, Frankfurt am Main, Germany; 4 Institute of Sport Science, University of Oldenburg, Oldenburg, Germany

**Keywords:** academic achievement, age at school entry, attention, cognitive abilities, relative age

## Abstract

**Introduction:**

Previous studies showed that children who are born at the start of the academic or sporting year achieve better examination results, on average, than children who are born at the end of the academic year. However, it is still unclear, whether the influence is related to relative age or to the age at school entry. While some findings report evidence for lower levels of language competence and academic progress for younger pre-school children in the first year of school, other results showed, that children who are born later in the academic year obtained lower educational attainment than their peers born at the beginning of the academic year.

**Methods:**

To examine these various assumptions, a cross-sectional study of relative age effects (RAE) on cognitive abilities in second and third graders from 25 different elementary schools (*N* = 583; *M*_age_ = 7.68, *SD* = 0.67) was conducted.

**Results:**

A set of analyses of variance (ANOVAs) revealed no associations between relative age and cognitive measures. However, results showed that children who are enrolled by their parents early in school obtained higher achievements for all cognitive variables than children of the same age, who were enrolled normally in school one year later.

**Discussion:**

These findings will be discussed under different aspects of schooling effects and the parents' motivations for early or late schooling as well as the implications for educational practice.

## 1 Introduction

Educational systems in developed nations utilize selection date policies to organize youth into cohorts (Wattie et al., 2012). Typically, a child must turn a specific age before a selection date (e.g., children who have reached the age of six by the 1^st^ of July) to be eligible to enter a specific cohort or group of youth (i.e., to enter school or to play on specific age-based sport team). In these contexts, youth born in the months following the selection date will be older than their peers born in the months prior to the selection date. These age differences within cohorts are described as relative age. The outcomes associated with relative age differences are RAE. Typically, relatively older youth, due to a fortuitous or purposefully planned birthdate, experience a range of advantages over their relatively younger peers (Dixon et al., 2020; Schorer et al., 2013). A considerable body of research has been dedicated to the examination of variations in selection age grouping policies, with a particular focus on the extant literature pertaining to sports-related relative age (Wattie et al., 2015; Steingröver et al., 2017a,b). The objective of this study was to examine RAE within an educational context. The aim of this study was to look beyond sports on this topic.

Research on RAEs in youth educational contexts consistently demonstrates advantages for relatively older members of cohorts, in both males and females (Bell and Daniels, 1990; Massey et al., 1996; Donofrio, 1977). Relatively older pupils are more likely to attain higher grades than their relatively younger peers across a range of different subjects (Massey et al., 1996; Donofrio, 1977). For example, Cobley and colleagues (Cobley et al., 2009) observed that the top 20% of achievers across all subjects (based on a composite attainment score from their grades from all subjects) were approximately four and a half times more likely to be relatively older rather than relatively younger. Similarly, Bedard and Dhuey (2006) examined data from the 1995 and 1999 Trends in International Mathematics and Science Study (TIMMS) from 19 countries of the ‘Organisation for Economic Co-operation and Development' (OECD; www.oecd.org) countries. Overall the results confirm previous research findings on relative age and academic achievement, with a 4–12 percentile disadvantage for the relatively youngest among 4^th^ grade children (approximately 9 years of age) and a 2 to 9 percentile difference for relatively younger 8^th^ grade children (approximately 13 years of age).

In addition to educational achievement, RAEs have been observed for school attendance rates (Cobley et al., 2009; Carroll, 1992), referrals for psychological counseling due to academic and/or behavioral problems (Drabman et al., 1987; Tarnowski et al., 1990; Thompson et al., 2004), and risk of psychiatric disorders (Goodman et al., 2003). In each case, relatively younger youth experience disadvantages and/or are more likely to experience negative developmental outcomes than their relatively older peers.

Wattie et al. (2015) argued that it is only possible to understand RAEs as the result of interactions between individual constraints (characteristics of the youth), task constraints (the characteristics of the activity), and environmental constraints (i.e., selection date policy and developmental environment). Moreover, Schorer et al. (2020) emphasized the need to observe RAEs over time based on their developmental characteristic. Indeed, relative age itself is the interaction between a person's date of birth (individual constraint) and the particular selection date policy (environmental constraint) used in a specific context. One important development in the study of RAEs has been the acknowledgment that slight variations in selection date policies and age group structures are salient environment constraints on RAEs.

### 1.1 Assigned and observed relative age

**Figure 1:**
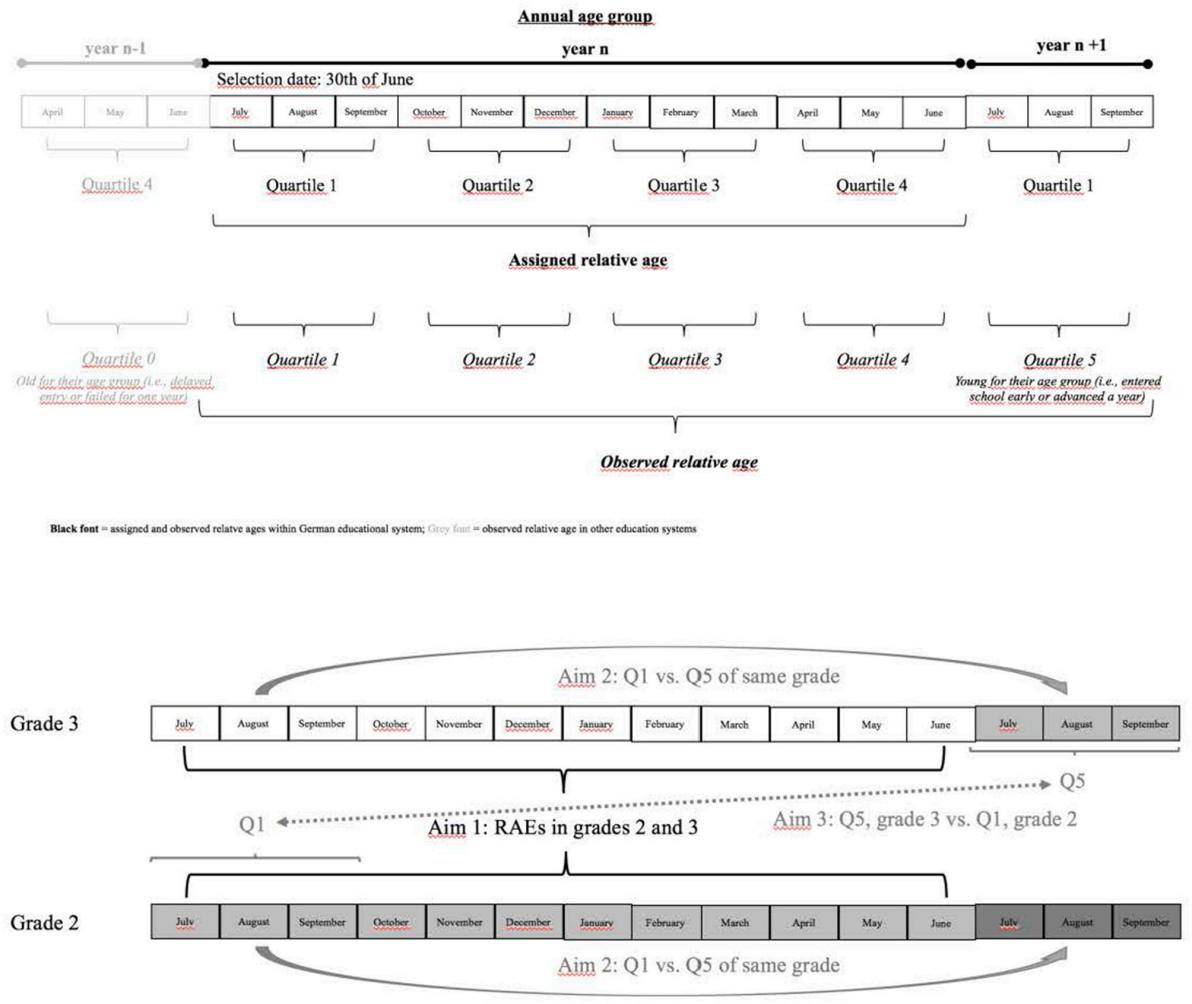
Annual age group: assigned and observed relative age.

In some education systems, selection date policies are rigidly enforced. In other systems, precocious youth may be permitted to enter into school earlier, and/or youth can “red shirt” and delay their entry into education so that they are not disadvantaged by their relative age. The result in such contexts is that there can be a difference between a youth's *assigned* relative age and their *observed* relative age (Bedard and Dhuey, 2006). For example, in a system with a selection date of June 30^th^, a child with a July birthdate (born in calendar year n + 1) would have an assigned relative age of Quartile 1 (i.e., relatively older). However, if allowed to enter education early, that child's *actual* relative age would be *younger* than the assigned Quartile 4-born youth (born in calendar year n) within the cohort that they find themselves within. In this case, we would observe a fifth quartile for this child (Q1^n+1^ = Q5). Figure 1 illustrates the dynamics between assigned and observed relative age.

Under different systems (assuming both use the same selection date to define their annual age groups) two children with the same date of birth could be the same *assigned*-relative age (i.e., the relative age that a child *should be* based on the selection date and their date of birth) but not necessarily the same *observed*-relative age (i.e., their relative age compared to those in the cohort they find themselves in). To date, while research in the domain of sport has considered variations in entry policies and the distinction between assigned and observed relative age, these particular constraints on RAEs in education have not been explored to the same extent. Therefore, the present study examined whether assigned and observed relative age, as well as early or late school entry, influence standardized cognitive performance in primary school students.

### 1.2 Research aims and objectives

The present investigation was guided by three overarching objectives. First, the relative age effect in education is tested based on three cognitive abilities: selective attention, information processing speed, as well as reasoning and analytical thinking (basic cognitive abilities). While in a conventional approach only four quartiles would be compared, in the German school system a fifth quartile needs to be considered. Children born in the first quartile (e.g., July to September, born in year n +1) are permitted to enter school early with an older cohort (i.e., born in year n). This option is selected by parents in discussion with the kindergarten teachers and is recommended if the perceived individual development of the child is advanced enough to warrant early entry to school.

Given that the relative age difference between all quartiles remained the same distance, we hypothesized that differences between quartiles [Q1^n^, Q2^n^, Q3^n^, Q4^n^, and Q5 (i.e., Q1^n+1^)] should be present for all cognitive abilities.

The second and third aim of the present study was to test for differences in children that entered school early. These “early joiners” (Q5) can be compared to Q1 children that are from the same assigned relative age within the same grade, which results in an age difference of approximately 1 year (second aim). Alternatively, they can be compared to the children that are the same age, the same assigned relative age, but entered school 1 year later as Q1, which results in a younger grade (aim 3). Both comparisons allow an insight into the role of selection by parents (aim 2) or duration of schooling (aim 3). Given that the “late remaining” children are most probable later in their development or skills than the “early joiners”, no clear hypotheses were possible. Therefore, these analyses are exploratory.

## 2 Materials and methods

### 2.1 Schools and participants

A total of *N* = 583 children (279 male, 304 female) with a mean age of 7.68 years (*SD* = 0.67) participated in this study. The children were recruited from 25 primary schools located in three different federal states of Germany (North Rhine-Westphalia; Hesse, and Lower Saxony). Due to the cross-sectional group design of the present study, children had already been assigned to school classes prior to this investigation. According to Elder and Lubotsky (2009), we measured families' household income, mother's and father's educational level, cultural practice, extra-curricular learning, as well as school grades for social behavior and oral collaboration to control for systematic influence on the dependent variables.

The study was approved by the institutional review board of the university of Frankfurt am Main in Germany. Additional written informed consent was obtained from school administration and parent. In addition, a verbal consent regarding participation in the study was obtained from the children. This study was part of a larger research project on the effects of music and natural science training on cognitive and emotional skills funded by the Federal Ministry of Education and Research (*No. 01KJ0807)*.

### 2.2 Measurements

The relationship between cognitive abilities and academic achievement across schooling is well observed and various psychological constructs have been analyzed as predictors of academic success, such as executive functions, IQ, information processing speed and attention (Rohde and Thompson, 2007; Deary et al., 2007; Rindermann and Neubauer, 2004). Information processing speed is a key predictor of higher-order cognitive characteristics (i.e., fluid intelligence, working memory, and number sense), which in turn contribute to overall academic success (Tikhomirova et al., 2020). This correlation between information procession speed and academic achievement is particularly pronounced among primary school children (Rindermann and Neubauer, 2004). In addition, selective attention—the ability to highlight relevant information while successfully managing distractions—plays a crucial role in learning processes and has been identified as an important predictor of academic performance in children and adults (Stevens and Bavelier, 2012; Ahmad and Sultana, 2021; Roden et al., 2014).

When considering the influence of relative age on developmental outcomes, research has observed discrepancies between objective standardized measures and more subjective measures (Drabman et al., 1987). As such, it is important to consider objective and validated measures of cognitive abilities to accurately understand the influence of assigned and observed relative age effects. A detailed description of the standardized cognitive measures employed in this study is provided in the following sections.

#### 2.2.1 Basic cognitive abilities

The German adaptation of Thorndike's Cognitive Abilities Test, Primary II/Form 1 and 2 (Thorndike et al., 1974) by Heller and Geisler (1983) was administered to assess basic cognitive skills using its subtests number 3 and 4: reasoning and analytical thinking. According to Heller and Geisler (1983), these subtests correlate highly with the total score of all subtests (*r* = 0.78 for subtest 3 and 0.86 for subtest 4). Results were assessed using raw data and aggregated for the two subtests.

#### 2.2.2 Selective attention

The d2 test of selective attention (Brickenkamp, 1962) is a graphical crossing-out test. During the test, participants are asked to identify targets within sequences of the letters “d” and “p” by marking (crossing out) the target with a pencil. Each letter has one to four vertical line marks in its immediate vicinity in subscripts and/or superscripts. Only “d” accompanied by exactly two line marks (two above, two below, or one above and one below the letter) have to be marked. The test has 14 rows of 47 letters in each row, thus totaling 658 letters. Participants are given 20 s for each of the 14 rows of the test. Total testing time is 4 min and 40 s. Before the test started, standardized explanations for children were read out from the manual and questions were answered by trained administrators. In addition, a practice row with 47 letters was performed by the participants and after examining each participant's practice row for correctness, the test started. Attention measures from the test are the total rate of processed letters (GZ-value for each row and in sum), different types of error values (F-value) and the concentration performance (KL-value for each row and in sum) representing the final selective attention score. For our purposes, only the KL-values were used for data analyses. The value consists of the number of correct letters crossed out minus the number of falsely marked letters. The d2 test of attention correlates highly with other measures of attention and is well validated. The retest reliability for KL is *r*_tt_ > 0.90. For children between 9 and 10 years of age, Cronbach's alpha level for KL is 0.93 (Brickenkamp, 1962).

#### 2.2.3 Processing speed

The “Zahlen-Verbindungs-Test, ZVT” (Oswald and Roth, 1987) is a reliable measure of information processing speed, which correlates highly with standard psychometric tests of intelligence such as the Wechsler, Raven or the Culture-Fair-Intelligence-Test. Participants are requested to connect 90 digits in each of four matrices A, B, C and D. Digits are dispersed across a sheet of paper in consecutive order from 1 to 90. The spatial distance between consecutive numbers is kept constant, and the amount of information in every matrix is about 136 bit. This means that the possible alternatives for each choice to connect one digit with another is equal across the four matrices. Participants work for exactly 60 s on each of the matrices. Total testing time is four min. Standardized explanations for group tests were read out from the manual, and possible questions were answered by our trained administrators. Two small matrices for practice with digits from 1 to 20 were performed by the participants just to make sure that everyone understood the instructions. Composite processing speed scores were measured via the performance in bit/seconds for each correctly attained digit. This means that the more digits were correctly linked in one matrix (up to the maximum of 90), the higher the performance for processing speed was. The retest reliability for the ZVT for group settings is *r*_tt_ = 0.81, and the retest reliability for the single matrices ranges from *r*_tt_ = 0.88 to *r*_tt_ = 0.93. Furthermore, the ZVT correlates at medium to high levels (from *r* = 0.69 to *r* = 0.80) with standard psychometric tests of intelligence (Oswald and Roth, 1987). In addition, the test places minimal demand on memory and attention by minimalizing concurrent processing and/or attention shifts, which automatically marginalizes the demand on other cognitive skills such as working memory. Moreover, differences in performances on the processing speed task can be attributed to differences in the speed with which decisions are made rather than the time required for motor response execution (Rammsayer and Stahl, 2007).

### 2.3 Procedure

The study was designed as a cross-sectional group study in which primary school children were examined for two consecutive days. Participants were tested in group settings in their classrooms. Basic Cognitive abilities (KFT), selective attention (d2) and information processing speed (ZVT) performances were embedded in a larger research protocol with standardized and demographic questionnaires. On the first day, children completed the selective attention and processing speed tests. On the following day, children accomplished subtests 3 and 4 of the cognitive ability test. Instructions were given in German. Trained test administrators carried out the assessments according to the instructions of a detailed manual and script. Measurements at schools were conducted within the first 3 weeks of the school year.

We used standardized questionnaires to collect data on demographic and socioeconomic variables. Items were taken from the Progress in International Reading Literacy Study (PIRLS) questionnaire (Bos et al., 2005). This information was obtained through questionnaires and telephone interviews with parents as part of a larger study. It was assessed using a Rasch-scaled composite score of 14 variables, including parental education, income, and cultural practices. Furthermore, class teachers provided feedback about social behaviors and oral collaboration via school marks.

### 2.4 Data analysis

According to a pre-study power-analysis conducted using G^*^Power (Buchner et al., 1997) this sample size was considered sufficient to observe small effects (*f* = 0.20) in a fixed effects, one way design (α:0.05, power (1-β):0.80, correlations between repeated measures: *r* = 0.50). The selected effect size of *Cohen's f* = 0.20 is situated between the range designated for small effect sizes (*f* = 0.10) and medium effect sizes (*f* = 0.25) (Gignac and Szodorai, 2016). We focus on a relative small effect size in our power analyses since evidence on RAE on cognitive variables like IQ, information processing speed or selective attention of preschool children are rarely reported.

Whilst the majority of the studies cited above used similar regression or ANOVA analyses, including *post-hoc* analyses with adjusted *p*-values to control for type 1 errors resulting from multiple comparisons, the same statistical procedures were also employed in the present study. Analyses of variance (ANOVA) were conducted for the independent measures of the parental income, mother's and father's educational level, cultural practice, social behavior and oral collaboration. Preconditions for conducting ANOVAs were tested (normality, homogeneity of variance). Moreover, gender and migration background for second and third graders were recorded.

The further analyses for the dependent variables were divided in three sections. First, the results for the d2 are presented. In a second part, the findings for the ZVT are offered. And in a third step, the analyses for the KFT are presented. All three results sections follow the same logic and differentiate between second and third grades pupils. First, the differences between quartiles are examined (hypothesis 1). In a second step, comparisons between quartile 1 and quartile 5 are drawn to investigate hypothesis 2. Thirdly, quartile 5 of grade 3 and quartile 1 of grade 2 are compared to check for differences between groups with varying schooling lengths but the same age (hypothesis 3). All analyses were conducted with SPSS version 30.

## 3 Results

### 3.1 Results for independent measures

Independent measures of parental income, mother's and father's educational level, cultural practice, extra-curricular learning, social behavior and oral collaboration did not significantly differ (Supplementary Table 1) between quartiles for second graders (all *F*-values < 2.23, all *p*-values > 0.09) and third graders (all *F*-values < 1.49, all *p*-values > 0.22), except the following: Children from the second class showed a significant difference for the oral collaboration grades, *F*(3, 207) = 2.78, *p* = 0.04. However, *post-hoc* test for multiple comparison of means with Scheffé adjustments revealed no significant difference between the quartiles. Moreover, gender and migration background were well balanced between the quartiles for second graders (gender: χ^2^ = 1.06; *df* = 3; *p* = 0.79; migration background: χ^2^ = 8.34; *df* = 6; *p* = 0.21) and third graders (gender: χ^2^ = 3.20; *df* = 3; *p* = 0.36; migration background: χ^2^ = 3.19; *df* = 6; *p* = 0.78).

Given the absence of statistically significant disparities among the quartiles with respect to gender, migration background, age, parental income, educational level, cultural practice, and grades for social behavior and oral participation, these independent variables were excluded from the subsequent analysis of the dependent variables.

### 3.2 Results for selective attention (d2)

The majority of studies in the field of RAE have utilized one-sided testing procedures, as relatively older people generally exhibit superior performance in comparison to younger individuals (Cobley et al., 2009). Consequently, the decision was taken to investigate relative age effects with four quartiles by one-tailed univariate analyses of variance. We found significant differences for grade 2, *F*(3, 287) = 3.41, *p* < 0.01, *f* = 0.15, as well as for grade 3, *F*(3, 168) = 2.22, *p* = 0.04, *f* = 0.15. *Post-hoc* tests with the Scheffé-procedure revealed differences between quartiles 3 and 4 for the second graders (*MD* = 13.31, *p* = 0.05). However, *post hoc* analyses revealed no statistically significant differences among third graders (all *p*-values > 0.15).

Comparing the performances of quartile 1 and quartile 5 of the same grade, *t*-tests for independent samples revealed neither differences for grade 2, *t*(105) = 0.57, *p* = 0.57, *d* = 0.12 nor for grade 3, *t*(56) = 0.90, *p* = 0.37, *d* = 0.24.

**Figure 2:**
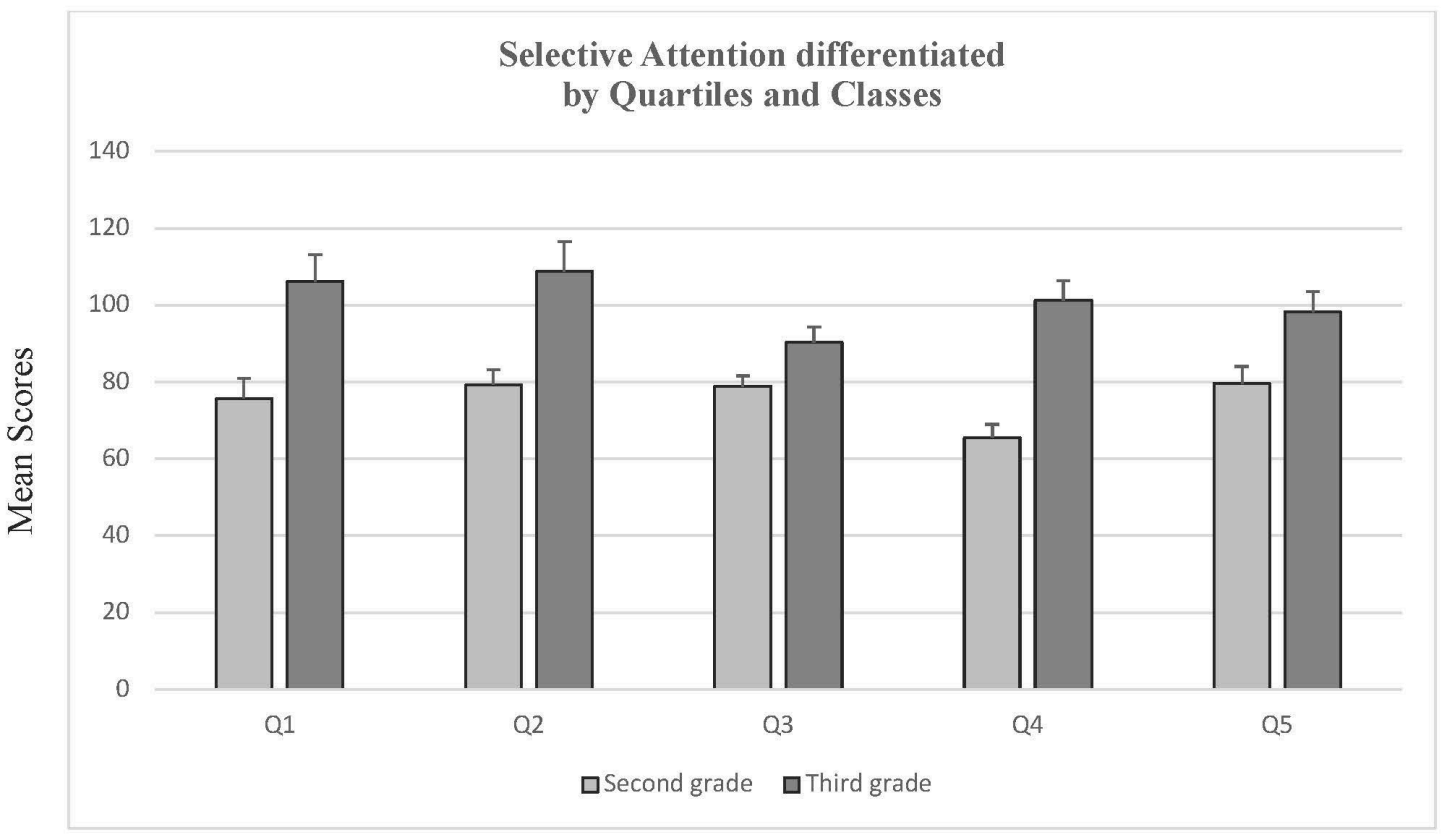
Selective attention differentiated by quartiles and classes. Means and standard errors (SEM) of d2- test performances (KL) differentiated by grades and quartiles.

To be able to compare pupils of the same age, but with a difference of 1 year in schooling we matched the pupils in quartile 1 from second grade with the ones from quartile 5 from third grade. A *t*-test for independent samples revealed significant differences, *t*(82) = 2.75, *p* < 0.01, *d* = 0.66. As can be seen in Figure 2, the pupils from quartile 5 in grade 3 outperformed the ones from quartile 1 in grade 2.

### 3.3 Results for information processing speed (ZVT)

To check for differences between the four quartiles, we ran a one-tailed analysis of variance (see Figure 2). For grade 2, there were significant differences, *F*(3, 288) = 2.59, *p* = 0.02, *f* = 0.14, but not for grade 3, *F*(3, 170) = 1.11, *p* = 0.17. *f* = 0.14. No *post-hoc* differences could be revealed with the Scheffé-procedure (all *p*-values > 0.361).

**Figure 3:**
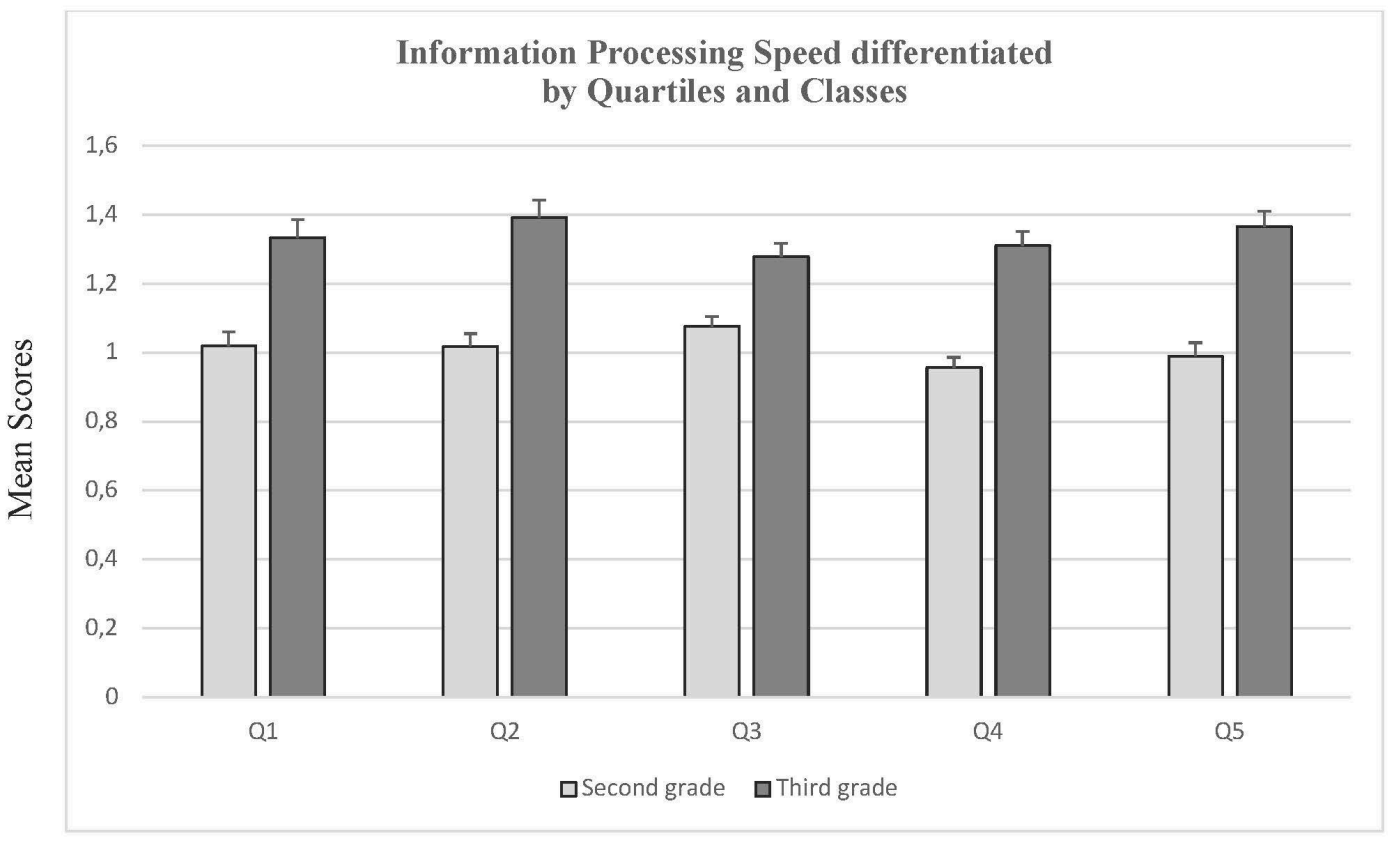
Information processing speed differentiated by quartiles and classes. Means and standard errors (SEM) of processing speed- test performances differentiated by grades and quartiles.

Comparing quartiles 1 and 5 from the same grades, neither differences for grade 2, *t*(105) = 0.57, *p* = 0.57, *d* = *0.1*1 nor for grade 3, *t*(58) = 0.47, *p* = 0.64, *d* = *0.1*2 were revealed. For the comparison of grade 3 pupils from quartile 5 and the ones from grade 2 quartile 1, however, significant differences could be demonstrated, *t*(82) = 5.31, *p* < 0.01, *d* = 1.26. As can be seen in Figure 3, the grade 3 quartile 5 pupils outperformed the grade 2 quartile 1 pupils.

### 3.4 Results for basic cognitive abilities (KFT)

In the first step, a comparison between quartiles 1 to 4 were drawn for basic cognitive abilities. A one-tailed univariate analyses of variance revealed significant differences for grade 2, *F*(3, 274) = 3.02, *p* = 0.02, *f* = 0.16, but not for grade 3, *F*(3, 164) = 0.08, *p* = 0.48, *f* = 0.04. *Post-hoc* test with the Scheffé-procedure revealed differences between quartiles 3 and 4 for the second class, *MD* = 2.50, *p* = 0.03.

**Figure 4:**
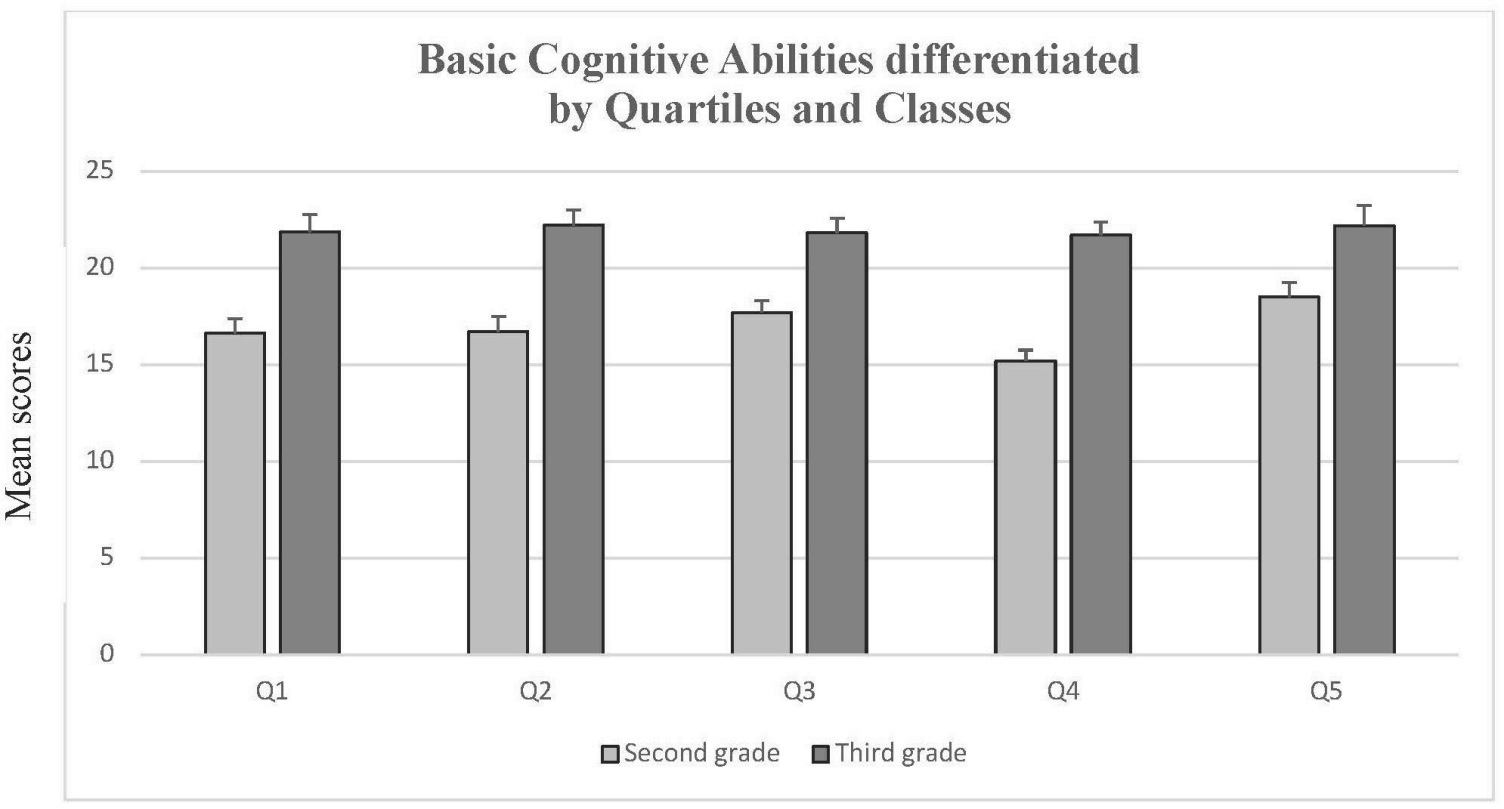
Basic cognitive abilities differentiated by quartiles and classes. Means and standard errors (SEM) of cognitive basic abilities- test performances differentiated by grades and quartiles.

No significant differences were observed between quartile 1 and 5 from the same grade, neither for grade 2, *t*(99) = 1.79, *p* = 0.08, *d* = *0.3*6, nor for grade 3, *t*(52) = 0.24, *p* = 0.81, *d* = *0.0*7. But—as with the other two dependent variables—there were significant differences between the pupils with the same age but different schooling durations, *t*(74) = 4.39, *p* < 0.01, *d* = 1.05. Again, the grade 3 quartile 5 pupils outperformed the grade 2 quartile 1 ones (cf. Figure 4).

## 4 Discussion

This study investigated the influence of early entry to school on cognitive abilities for 2^nd^ and 3^rd^ graders. First, classical relative age effects in education were examined for cognitive abilities, such as selective attention, information processing speed, and basic cognitive abilities. Second, we examined whether early school joiners (Q5) differ from children that are from the same assigned relative age within the same grade (Q1) concerning their cognitive outcomes. The third aim of the study was to compare the children that are the same age, the same assigned relative age, but entered school one year later (Q1, grade 2), which results from children in a younger grade (Q5, grade 3). According to discrepancies between objective standardized measures and more subjective measures with respect to the RAE, only standardized test inventories were used to measure cognitive performances.

Concerning our first aim, our results did not show the expected relative age effects on any cognitive abilities. Additionally, significant differences between the quartiles for the observed cognitive abilities were only reported in the second grade showing rather small effect sizes. This is in contrast to previous research on RAEs in youth educational contexts, that consistently demonstrates advantages for relatively older members of cohorts. For example, relatively older pupils are more likely to attain higher grades than their relatively younger peers across a range of different subjects (Massey et al., 1996; Cobley et al., 2009; Bedard and Dhuey, 2006). However, in a longitudinal study of regularly enrolled children—children who enrolled in school according to the cut-off date regulation—by Gold et al. (2012), the authors examined the effects of relative age at school enrolment on academic achievement, by holding the length of school attendance constant. For comparison, the performance of children enrolled early and late in school was reported in a separate analysis. Indicators of academic achievement, reading and spelling skills were recorded at the end of 1st, 2nd, and 3rd grade, and basic cognitive abilities were assessed shortly after school enrolment as a moderator variable via a German adaptation of Cattell's *Culture Fair Intelligence* test by Weiß and Weiß (2006). Results showed no RAEs between the oldest and youngest children in the group of regularly enrolled children in terms of cognitive abilities, gender and migration background (family language). They also did not differ in terms of academic achievement growth for the majority of measured skills. Finally, in the third grade, no difference in performance between the two age groups in any of the competency areas were observed. Therefore, the authors suggested that relative age effects may be a temporary phenomenon with regard to the development of academic performance.

There could be varying explanations for the absence of any relevant RAEs in the present study. First, most of the reported literature in the field of RAEs in education focus on education outcome variables, like school grades or test results for class works, whereas the present study used standardized measures of cognitive abilities, which are only indirectly related to school achievement outcome variables. Second, some previous studies used self-report measures while we have used objective measures. This variation in assessment strategies might have led to the discrepancies in the observed RAEs (Thompson et al., 2004). Taken together, previous studies mainly focused on RAEs on self-reports or academic skills whereas the present study focused on cognitive abilities like IQ, attention and processing speed. This methodological discrepancy might explain why we did not observe RAEs in our findings. Moreover, some studies may not have taken into account that enrolment cohort also included children who were enrolled early and late, which can lead to a systematic confounding of the RAE effects with ability effects (Thorndike et al., 1974).

Regarding our second and third research aim, we wanted to investigate if actual age (aim 2) or school duration (aim 3) play a stronger role for cognitive performances. For the second research aim, we could not observe any benefits for the “early joiners” compared to the school children in quartile 1 in the same assigned relative age for any cognitive performances.

For the third aim of the present study, third graders from quartile 5 outperformed second graders from quartile 1 in all cognitive variables. These findings to some extent contradict previous research on RAE in education, which argues the effects are primarily due to maturational and developmental differences associated with *age* differences. These assumptions are based on the notion that older children in a cohort have more cognitive, motivational, and emotional (as well as physical) skills necessary for learning in school than younger children because of their more advanced biological development. However, whether schooling duration has a substantial influence on the development of cognitive abilities, especially IQ, is an ongoing debate in the field of educational and developmental psychology. For example, Ceci (1991) reviewed the evidence from various sources that might have substantial influence on the development of intelligence, like the high correlations between IQ and the amount of time in school or the effects of intermittent school attendance, delayed onset or early termination of school on intelligence. He strongly suggested a causal relationship between schooling and IQ that is independent of parental motivation or socioeconomic status. He further argued, that IQ should be perceived “largely as a measure of direct and indirect school learning” (Ceci, 1991). However, most of his reviewed studies based on correlational research rather than (quasi-) experimental studies. Further studies with quasi-experimental designs tried to separate the effect of schooling from the effect of chronological age on the growth of cognitive abilities (Ceci, 1991; Cahan and Cohen, 1989; Stelzl et al., 1995). Stelzl et al. (1995) showed that school effects had a considerable impact on crystallized and fluid intelligence, independent of chronological age. Moreover, schooling effects covered almost all the progress made in the Culture fair intelligence test (CFT 20) and in the vocabulary subtest of the Wechsler Intelligence Scale for Children (Wechsler and Kodama, 1949) at the age level of 10-year-old primary school children. The authors concluded that schooling influences the development of general intelligence because it challenges children cognitively. These findings contradict the assumption that the development of fluid intelligence is primarily based on biological processes of neural growth and maturation, and is not influenced by formal education (Cahan and Cohen, 1989).

In similar study designs researchers have observed that outcomes such as mental arithmetic are more related to amount of school and not age (Bisanz et al., 1995). Going forward it will be important to distinguish characteristics which are related to age as opposed characteristics related to the amount of time in school (Bisanz et al., 1995). Furthermore, in addition to relative age differences and amount of schooling, school entrance age (Bisanz et al., 1995; Black et al., 2011; Datar, 2006) may also need to be considered when trying to understand developmental and cognitive outcomes.

To explain the present findings, several hypotheses might be put forth. First, selection effects may have distorted the outcome of our analyses, as parents' motivations for early or late schooling are generally closely related to considerations of a child's school readiness and to the expected school performances. Correspondingly, it could be assumed that among the youngest group of the regular school children, those children are missing who were later enrolled to school should be due to a lack of schooling ability. Otherwise, it could be assumed, that in the group of older children among the regularly enrolled children those children are missing, that were enrolled prematurely due to their excellent achievement potential (Datar, 2006). Both of these interacting selection effects would lead to an underestimation of the observed RAEs, as the performance of the youngest would be overestimated, whereas the performance of the elders would be underestimated. Unfortunately, we cannot test such a selection bias in our current sample, because the pupils have already been in school for at least 1 year. Future research might look at children in the last preschool year, in which the decision to start school is made for Q5 kids.

Secondly, one could argue that children who entered school 1 year earlier might achieve higher cognitive performance, compared to children of the same age, due to their longer duration of schooling. Those benefits represent schooling effects that are well examined in the existing literature (Elder and Lubotsky, 2009; Ceci, 1991; Black et al., 2011; Datar, 2006; Ceci and Williams, 1997). However, research focussing on RAE on cognitive abilities showed a more differentiated picture: while some studies report benefits of schooling effects on cognitive outcome variables like IQ, processing speed, working memory and attention in kindergarten and preschool children (Wang et al., 2016; Burrage et al., 2008) others do not (Jonsson et al., 2017). For example, Wang et al. (2016) showed that the combined effects of schooling and age for primary school children were about five intelligence points per year. This means, that the schooling effect was six times higher than the age effects. In contrast, Jonsson et al. (2017) did not find any differences for schooling effects on processing speed, working memory, inhibition, or attention measures in primary school children.

### 4.1 Limitations

This study is limited in several ways. Firstly, the study is based on a cross-sectional design observing groups in their natural environment at school. Although we evaluated demographic variables and educational achievement such as educational level, social behavior, oral collaboration, to determine any systematic effect, interpretations must be made with caution. Secondly, we did not measure the exact date of testing, which in relation to the date of birth would offer a more precise calculation of RAEs. Third, we cannot say whether those observed effects are sustainable over time or if they would disappear during the period of primary school. There is only little evidence from longitudinal studies, suggesting that initial performance advantages for reading skills in relatively older children at school entrance diminishes in the second grade and are no longer significant in the third grade, if the authors controlled the RAEs for IQ (e.g. Gold et al., 2012). Hence, further longitudinal study designs are necessary to examine the sustainability of assigned and observed RAE's on specific and global cognitive abilities in primary school children.

Finally, we want to address concerns regarding the statistical analyses. Whilst the majority of the cited studies utilized similar regression or ANOVA analyses, including *post-hoc* analyses with adjusted p-values to manage type 1 errors arising from multiple comparisons, the results of the present study must be treated with caution, insofar as they have the capacity to elevate the type 1 error rate as a consequence of the nested data structure (Theobald et al., 2019; Bono et al., 2021). Further studies might suggest the use of General Linear Models (GLM), Generalized Estimating Equations (GEE), or Structural Equation Models (SEM) to examine RAE in educational settings.

To put it concisely, this study is the first to look at the effect of the possible early entry into school in the German education system. In contrast to other studies in education, we did not find the expected RAEs. The strongest differences were revealed between early entering pupils and their age-matched counterparts from grade below. Both hypotheses, selection and schooling duration, could explain at least parts of our results. Most probable an interaction of these two mechanisms might explain most of the observed variance. While selection effects in this sample might play some role, the effects of the schooling duration seems to be bigger for their performance in basic cognitive tasks. This is in accordance with previous studies by Gold et al. (2012) and Thoren et al. (2016) on RAEs in pupils in Germany.

### 4.2 Implications for educational practice

Parents may find the results of the present study helpful in their decision making about earlier school entrance. Parents' biggest concerns about school entry are related to its short-term and long-term influence on pupils' academic achievement and social–emotional development. Based on their personal experiences, parents and pupils can understand and appreciate the short-term benefits of earlier enrolment. However, they are uncertain or unconvinced about the long-term effects of earlier enrolment, or whether there are any benefits at all in the long run. Therefore, evidence on the long-term effects of an educational intervention must ultimately be based on longitudinal/retrospective research studies. However, such studies are few and far between. The results of the present longitudinal study can provide at least limited information about early entering pupils and their age-matched counterparts from grade below. Specifically, school duration seems to have a stronger effect than the actual age of 3^rd^ and 2^nd^ graders. Those findings support the results from other longitudinal studies, like Gold et al. (2012), that reported a better academic performance development of children enrolled early in school compared to children enrolled late. These findings were also evident in the second and third grades.

The study of the relative age effect (RAE) on the development of cognitive and emotional abilities in schoolchildren has gained importance worldwide in recent decades, driven by the implementation and modification of public policies aimed at improving teaching and learning processes (Urruticoechea et al., 2021; Pehkonen et al., 2025). Furthermore, the effects of relative age on educational development, particularly through the creation of grouping systems by public policies, should also be considered in the future, as these can influence cognitive development (individual constraints) and socio-emotional development (environmental constraints). Therefore, it seems essential to observe and consider the long-term consequences of RAE in education in order to determine the importance of relative age for the holistic development of the individual.

Although we did not find any significant RAEs in our study, these findings cannot be used to justify delayed school entry. Delayed school entry “postpones” the opportunity for systematic learning, which is particularly detrimental for children from adverse family learning environments (Elder and Lubotsky, 2009). Moreover, other studies have shown that primary schools tend to teach in a performance-balanced manner, suggesting that children's different learning backgrounds and performance are not necessarily a risk factor for future learning development—which may ultimately serve as further evidence to reduce concerns about earlier school entry.

## 5 Conclusions

No RAEs on cognitive performance were found in the present study. Given the RAEs on academic performance reported in other longitudinal studies also seem to be small and, gradually cancel out as primary school years progress (e.g. Gold et al., 2012), RAEs seem to have rather less influence on the development of academic performance or cognitive development in the long run. The findings of this study demonstrate that the duration of schooling, rather than age, exhibits a positive correlation with the cognitive development of primary school children.

## Data Availability

The original contributions presented in the study are included in the article/Supplementary material, further inquiries can be directed to the corresponding author.
