# Supplementary material for: Assigned vs. observed relative age: the association of early entry to school on cognitive abilities and its implications for educational practice
Source: Front Psychol. 2026 Feb 18;17:1708843. doi: 10.3389/fpsyg.2026.1708843 (PMC12957081; doi:10.3389/fpsyg.2026.1708843)
Supplement: Supplementary file 1 [file Table_1.docx]

Table 1: Mean values (and SD) of the independent variables for classes (2nd and 3rd) and quartiles (1–4).

|  | 2nd. class | | | | | | |  | 3rd. class | | | | | | | | | | | | |  |
| --- | --- | --- | --- | --- | --- | --- | --- | --- | --- | --- | --- | --- | --- | --- | --- | --- | --- | --- | --- | --- | --- | --- |
|  | *M (SD)* | | | | | | |  | *M (SD)* | | | | | | | | | | | | |  |
| *Measures* | Q 1 | Q 2 | Q 3 | Q 4 | *F-Value* | *df* | *p* |  | Q 1 | Q 2 | | Q 3 | | Q 4 | | *F-Value* | | *df* | | *p* | |  |
| parental income | 1.28 (1.37) | 1.23 (1.42) | 1.63 (1.65) | 1.68 (1.70) | 1.02 | 191 | 0.38 |  | 1.68 (1.57) | | 1.59 (1.73) | | 2.15 (1.48) | | 1.65 (1.70) | | 0.52 | | 106 | | 0.67 | |
| educational level (father) | 3.59 (2.14) | 3.47 (2.08) | 4.09 (2.00) | 3.57 (2.11) | 1.02 | 201 | 0.38 |  | 4.09 (2.07) | | 4.61 (1.50) | | 4.25 (2.14) | | 4.06 (1.82) | | 0.44 | | 116 | | 0.73 | |
| educational level (mother) | 3.14 (1.96) | 3.61 (1.97) | 3.56 (1.92) | 3.39 (1.97) | 0.53 | 215 | 0.67 |  | 3.77 (1.90) | | 4.63 (1.61) | | 4.15 (1.99) | | 3.81 (1.75) | | 1.20 | | 121 | | 0.31 | |
| cultural practice | 2.23 (0.99) | 2.37 (1.09) | 2.59 (1.16) | 2.38 (1.25) | 0.61 | 51 | 0.61 |  | 2.59 (1.37) | | 2.76 (1.27) | | 2.93 (1.07) | | 2.62 (0.98) | | 1.49 | | 162 | | 0.22 | |
| social behavior | 2.39 (1.26) | 2.04 (1.10) | 1.92 (0.98) | 2.22 (0.87) | 2.08 | 208 | 0.10 |  | 2.33 (1.20) | | 2.14 (0.76) | | 2.15 (0.93) | | 2.1 (0.91) | | 0.30 | | 131 | | 0.83 | |
| oral collaboration | 2.5 (1.18) | 2.56  (1.18) | 2.11 (0.96) | 2.62 (1.12) | 2.80 | 204 | 0.04 |  | 2.33 (1.02) | | 2.04 (0.88) | | 2.31 (1.24) | | 2.12 (1.01) | | 0.55 | | 130 | | 0.65 | |

*Note: Q 1-4 = quartiles quartiles for second and third graders; Parental income: 0 =< €20.000; 1 = €20.000–€29.999; 2 = €30.000 –€39.999; 3 = €40.000–€49.999; 4 = €50.000–€59.999; 5 =>€60.000; parents education level: 1 = did not go to school, no graduation; 2 = secondary modern school qualification (ISCED level 2); 3 = matriculation standard (ISCED level 3,4); 4 = university degree (ISCED level 5); cultural practice: 1 = less than 10 books at home; 2 = 11–25 books; 3 = 26–100 books; 4 = 101–200 books; 5=>500 books; social behavior (school notes): 1 = very good / excellent (grade 1), 2 = good (grade 2), 3 = satisfactory / average (grade 3); 4 = sufficient (grade 4);* oral collaboration (school notes): *1 = very good / excellent (grade 1), 2 = good (grade 2), 3 = satisfactory / average (grade 3); 4 = sufficient (grade 4).*
